# Psychometric properties of the German version of the Leicester Cough Questionnaire in sarcoidosis

**DOI:** 10.1371/journal.pone.0205308

**Published:** 2018-10-04

**Authors:** Jonas Christian Schupp, Urs Alexander Fichtner, Björn Christian Frye, Katja Heyduck-Weides, Surinder S. Birring, Wolfram Windisch, Carl-Peter Criée, Joachim Müller-Quernheim, Erik Farin

**Affiliations:** 1 Department of Pneumology, Faculty of Medicine, University Medical Centre, Freiburg, Germany; 2 Section of Pulmonary, Critical Care and Sleep Medicine, Yale University School of Medicine, New Haven, Connecticut, United States of America; 3 Institute for Quality Management and Social Medicine, Faculty of Medicine, University Medical Centre, Freiburg, Germany; 4 Division of Asthma, Allergy and Lung Biology, King’s College London, London, United Kingdom; 5 Department of Pneumology, Cologne-Merheim Hospital, Kliniken der Stadt Köln gGmbH, Witten/Herdecke University Hospital, Cologne, Germany; 6 Department of Sleep and Respiratory Medicine, Evangelical Hospital Göttingen-Weende, Bovenden, Germany; Hong Kong Polytechnic University, HONG KONG

## Abstract

**Background:**

Cough is one of the most common symptoms in general and pulmonary medicine with profound negative impact on health-related quality of life (HRQL). The Leicester Cough Questionnaire (LCQ) is a validated HRQL questionnaire, yet a validated German version of the LCQ is not available and it has never been tested in a cohort with sarcoidosis.

**Objectives:**

To translate the LCQ into German and determine its psychometric properties.

**Methods:**

The LCQ was translated in a forward-backward approach. Structured interviews in sarcoidosis patients were performed. Subsequently, sarcoidosis patients were asked to answer the German LCQ and comparative questionnaires. Distribution properties, item difficulty, concurrent validity, Rasch model fit and internal consistency of the German LCQ were determined.

**Results:**

200 patients with sarcoidosis were included. We provide evidence for reliability, unidimensionality and internal consistency. However, only a moderate correlation with general and respiratory-specific HRQL questionnaires, no Rasch model fit could be shown. Skewed responses caused by floor effects were detected.

**Conclusion:**

We demonstrate that the German LCQ is valid and reliable and its psychometric properties fulfil the standards required for its use in clinical settings as well as in interventional trials.

## Introduction

Cough is one of the most common symptoms in general and pulmonary medicine and has a profound negative impact on health-related quality of life (HRQL) in affected patients[1]. Patients with chronic cough suffer from physical illness, like chest pain, syncope and incontinence, and psychological morbidity, like social embarrassment and depression[2]. A myriad of diseases can cause chronic cough, common diseases like asthma or gastroesophageal reflux disease or rare diseases like sarcoidosis.

Sarcoidosis is a systemic granulomatous disease of unknown aetiology[3]. Patients with sarcoidosis typically suffer from pulmonary symptoms like cough, dyspnoea or chest pain with subsequent reduction of their general health status and their respiratory-specific HRQL[3–6]. Cough is a common symptom in patients with sarcoidosis, with up to 50% affected patients [6–8]. Bronchial involvement, which can be demonstrated histologically, is supposed to be causative for cough in sarcoidosis patients[9]. In sarcoidosis, endpoints of clinical studies differ either depending on organ involvements or on general sarcoidosis symptoms like fatigue[10]. As symptoms like cough are difficult to be quantified objectively and usually accompanied with psychological morbidity, patient-reported outcomes should be implemented, at least as secondary endpoint, in clinical trials. The inclusion of measurements of HRQL in clinical trials is thus recommended by the World Association of Sarcoidosis and Other Granulomatous disease (WASOG)[10].

The Leicester Cough Questionnaire (LCQ) is a validated HRQL questionnaire on cough with three domains covering physical, psychological and social aspect of chronic cough[11]. The minimal important difference of the LCQ has already been established[12]. The LCQ has been translated into several languages including Dutch, Korean, Lithuanian, Mandarin, Polish, Portuguese and Spanish, and validated in corresponding cohorts[13–22]. It has been validated in different diseases, like chronic cough itself, cystic fibrosis including in children, bronchiectasis and COPD, yet not in a sarcoidosis cohort[20,23–25]. Furthermore, a revised version, the LCQ-acute, was developed for patients with acute cough[26].

The LCQ was already applied in several clinical studies on chronic cough testing different drugs including azithromycin, esomeprazole and itraconazole[27–29], as well as in a positive clinical study proving that morphine is an effective antitussive in chronic cough[30]. At the moment, it is in use in several clinical trials like in azithromycin in chronic productive cough (ClinicalTrials.gov Identifier: NCT02196493), amikacin in acute exacerbation of bronchiectasis (NCT02509091) or dornase alpha after lung transplantation (NCT01952470).

Taken together, the LCQ has been translated in multiple languages and validated in several diseases and is utilized in several completed and ongoing clinical studies, yet a validated German version of the LCQ is not available and it has never been tested in a cohort with sarcoidosis. Therefore, we aimed to translate and validate a German version of the LCQ in sarcoidosis patients to provide an instrument for future multinational observational and interventional studies on chronic cough and in particular in sarcoidosis.

## Methods

### Translation

In order to translate the LCQ[11] into German and validate it in a German cohort a multistep forward-backward approach was chosen[31]: First, the English version of the LCQ was independently translated by five authors (EFG, JMQ, JCS, WW, CPC), merged to a preliminary version followed by a backward translation by a native speaker. Some suggestions of the original author (SSB) were implemented, and afterwards the conceptual and cultural equivalence was confirmed by him. This German pilot version was tested in structured interviews of ten patients with sarcoidosis. Minor, final modifications based on these structured interviews were implemented and documented.

### Subjects

200 consecutive sarcoidosis patients of at least 18 years of age diagnosed of sarcoidosis according to the consensus statement of three scientific societies (ERS, ATS, WASOG)[32] were recruited over 12 months in the outpatient clinic of the department of pneumology of the university medical centre Freiburg. We obtained a positive vote by the Ethics Committee of the Albert Ludwig University of Freiburg, Freiburg, Germany prior to the initiation of the study and it was registered in the German Clinical Trials Register (reference number DRKS00010072).

### Study procedure

The participants were asked the following questionnaires and measurements: the LCQ in German, the Short Form (36) Health Survey (SF-36)[33], the Borg dyspnoea scale (CR-10)[34] and the visual analogue scale for dyspnoea (VAS-D)[35]. Furthermore, patients answered structured questions on their nationality, sex, duration of the disease, days of sickness per year, highest educational certificate, employment and family status.

### Statistical analysis

Our approach follows the original publication of the LCQ and another validation paper in the Dutch context[11,24].

#### Distribution properties

To give descriptive information and distribution properties, nonresponse rates, item difficulty, skewness and kurtosis were computed. Items with inappropriately high item difficulty, skewness or kurtosis as well as items with ceiling or floor effects were determined. Regarding the classification of ceiling and floor effects, we follow the approach of McHorney & Tarlov, Varni et al, and Lin et al defining a percentage of 0 to 15% as small, 16% to 30% as moderate and more than 30% as substantial floor or ceiling effect [36–38].

#### Item difficulty

In order to check for item difficulty, we computed the item difficulty index [39] using the sum of squared item values divided by the sum of squared maximum values. The calculated index ranges from 0 to 1 and is sensitive to the variance of multiscale items.

#### Confirmatory factor analysis

We conducted a confirmatory factor analysis using IBM SPSS AMOS 24 software to confirm the item-factor-relationship. A three-factor solution (corresponding to the assumed three constructs of the LCQ) was tested, based on a Maximum Likelihood estimation. Model fit was evaluated using Chi^2^ goodness of fit test, the Comparative Fit Index (CFI)[40], Tucker–Lewis index (TLI)[41], root mean square error of approximation (RMSEA), and standardized root mean square residual (SRMR). A non-significant Chi^2^ test indicates that the assumed model fits well the data. CFI and TLI values > 0.90 are considered as indication of good fit. RMSEA values <0.10 suggest a moderate fit; values <0.05 are a good fit[40]. The SRMR value should be under 0.08[40]. Satisfactory model fit is assumed whenever at least three out of four parameters produce good values[42]. In order to improve model fit, we additionally test a modified model. In order to do this, modification indices were explored to suggest residual correlations entailing a substantial improvement of fit [43]. However, we considered only changes if they were theoretically plausible.

#### Item response theory (IRT) analyses

We used the 1-parameter logistic IRT model (Rasch model) for item response theory analyses (software: WINSTEPS version 3.68), as it is an effective tool for clinical applications[44]. Following Reeve et al.[45], we chose a partial credit model as it also provides stable parameter estimates for even small case numbers and furthermore because the response categories are not identical for all items [45,46]. Infit and outfit mean square residual statistics (Infit MNSQ, Outfit MNSQ) were used as goodness-of-fit statistics. Mean squares greater than 1.0 indicate underfit to the Rasch model, meaning that a part of the model’s variance lies outside the underlying factor concept. Mean squares less than 1.0 indicate overfit to the Rasch model, meaning that the data is more predictable than the model expects [47,48]. For survey rating scales a suitable item mean-square range from 0.6 to 1.4 is recommended [47]. Items with a poor infit or outfit were discussed. Additionally, we checked for disordered response categories in the items by calculating the ability means for each item in WINSTEPS [48]. Disorder might occur for example if respondents find it difficult to differentiate between the various response categories for a particular item [49].

#### Internal consistency

Cronbach’s Alpha was calculated. Values between 0.7 and 0.8 can be considered as good, values between 0.90 and 0.95 as excellent. Values higher than 0.95 indicate item redundancy [50,51]. Item-total correlation >0.4 indicates that the item is adherent to other items measured using the same latent construct [52]. Concurrent validity: To evaluate concurrent validity, we tested the hypotheses below:

1. As the Borg scale and the VAS Dyspnoea assess lung symptoms, we expect moderate (negative) correlations (0.30> = r>0.5) of these parameters with the LCQ scales.
2. We expect high correlations (r> = 0.60) between the LCQ subscale “Physical” and the SF-36 Physical component scales, as well as between the LCQ subscales “Psychological” and “Social” and the SF-36 Mental component scales.

## Results

### Structured interviews

A pilot German LCQ was field-tested in ten patients with sarcoidosis by structured interviews. No patients had problems with understanding or answering the questionnaire. Some minor modifications were made in the final German LCQ to improve the comprehensibility of the questionnaire.

### Patient characteristics

200 patients with sarcoidosis were included in this study. We identified 5 cases with complete missing values on the 19 LCQ items and 2 cases with missing values on more than 50% of the LCQ items. By removing those seven cases from the analysis we ended up with a sample size of 193 patients. 48.7% of the patients were females, the mean age was 52.9 (± 12.6) years (see Table 1). 33.9% of the patients were unemployed, and in 30.1% of the patients the educational level corresponded to that of primary school. The majority of the patients (83.7%) suffered from a chronic course of their disease with at least three years of illness duration.

**Table 1:** Patient characteristics.

Variable	N_max_ = 193
**Age M and SD**	52.91 (12.62)
**Sex N (%)**	
Female	93 (48.7)
Male	98 (51.3)
**Nationality N (%)**	
German	172 (92.0)
Other	15(8.0)
**Partnership N (%)**	
Yes	144 (79.6)
No	37 (20.4)
**Level of education N (%)**	
Elementary school	56 (30.3)
Secondary school	63 (34.1)
Polytechnic secondary school	2 (1.1)
Technical college qualification	13 (7.0)
University qualification	44 (23.8)
Other or no certificate	7 (3.8)
**Employment status N (%)**	
Employed	115 (66.1)
Unemployed	59 (33.9)
**Duration of illness sarcoidosis N (%)**	
< 1 year	11 (6.2)
1–2 years	18 (10.1)
3–5 years	60 (33.7)
6–10 years	49 (27.5)
> 10 years	40 (22.5)

Notes: Totals not adding up to N = 193 are the result of missing values. M = mean score, SD = standard deviation. The percentages refer to the raw values including missing values.

### Distribution properties

In sum, 172 patients (86%) completed all 19 LCQ items. Overall, all items show low non-response rates with the highest rate (3.1%) on item number 9 (see S1 Table). All but one (item 15) items show floor effects with more than 30% of responses in the extreme categories indicating that the observed population is quite healthy. Both Skewness and Kurtosis lie in the range of -1.95 to +1.95 for almost all items except for item number 12 and 17. In those two items, the Kurtosis is slightly higher than the cut-off point.

### Item difficulty

Item difficulty values are considered as good when they score between 0.2 and 0.8. Higher values indicate that items are too easy and lower values indicate that items are too difficult. All index values lie in this range except for items 12, 14 and 17, which have high values indicative for being too easy (see S2 Table). The highest index value is reached for item number 12 with 0.83.

### Item response theory (IRT) analyses

Items were tested for Rasch model fit. Infit and outfit values that were beyond acceptable cut-offs were detected for items 3, 4, 5, 9, 11, 15 and 16. Inspection of the ability means for each item demonstrate disordered response thresholds for items 1, 4, 9, 13, 14, 15, 17 and 18. Overall, the LCQ does not fit to the probabilistic distribution of item response theory.

### Confirmatory factor analysis

Table 2 shows model fit statistics as a result of the confirmatory factor analysis. The original model showed barely satisfactory fit (p<0.001, TLI = 0.88, CFI = 0.9, RMSEA = 0.11). As modification indices suggested that model fit would be improved if correlated error terms were added, we identified three error term correlations we consider as reasonable. Two items refer to insomnia (physical subscale) and general frustration (psychological subscale) which might highly correlate and have a joint component not entirely explained by the underlying factors. Two other items comprise both the term “sorrow” (psychological subscale) which might cause high correlations. The last two items (Social subscale) we correlated refer to the impact of cough on general life aspects. We modified the model accordingly so that the new model shows improved model fit with a decreased Chi^2^ value of 298.84 and satisfactory TLI and CFI values above 0.9. RMSEA decreased to 0.10. The Chi^2^ test still suggests no good model fit. However, Chi^2^ tests have been found to be too sensitive to large sample sizes [37,53,54]. Therefore, we rely more to the other applied model fit indices and consider the modified model as satisfactory.

**Table 2:** Global fit indices of confirmatory factor analysis.

	N	χ ^2^	df	p	χ ^2^/df	TLI	CFI	RMSEA	RMSEA 90% CI	SRMR
**Original CFA model** ^a^	172	477.94	149	< .001	3.21	0.88	0.90	0.11	0.10–0.13	0.04
**Modified CFA model** ^b^	172	398.84	146	< .001	2.73	0.91	0.92	0.10	0.09–0.11	0.04

Notes:

^a^ Though EFA-(PCA) analysis suggested a one-factor solution, we performed an original CFA with the three latent variables “psychological, physical, social”.

^b^ As modification indices suggested that model fit would be improved if correlated error terms were included, we added three theoretically plausible correlated error terms.

Abbreviations: CFI: comparative fit index, CI: confidence interval, df: degrees of freedom, RMSEA: root mean square error of approximation, SRMR: standardized root mean square residual, TLI: Tucker–Lewis index.

### Internal consistency

Indicators for internal consistency are represented in Table 3. Cronbach’s Alpha is good to excellent for all three subscales of the LCQ (Cronbach’s alpha ranging from 0.87 to 0.94). The Item-total correlation falls below the acceptable range for the subscale “Physical” (0.24 to 0.74). We found that Cronbach’s Alpha of 0.87 would not decrease if item 15 would be eliminated. However, the mean inter-item correlation coefficient values do not fall below 0.44 so that we conclude that no items should be eliminated. Nevertheless, it should be mentioned that the subscale “physical” was the one with the weakest performance regarding internal consistency.

**Table 3:** Internal consistency.

Scale and items	N	Items	Item-Total correlation	Mean Inter-Item correlation	Cronbach’s alpha
**Physical** Includes items 01, 02, 03, 09, 10, 11, 14, 15	181	8	0.24–0.74	0.47	0.87
**Psychosocial** Includes items 04, 05, 06, 12, 13, 16, 17	183	7	0.53–0.83	0.70	0.94
**Social** Includes items 07, 08, 18, 19	187	4	0.66–0.90	0.73	0.92

### Concurrent validity

As expected in hypothesis 1, we observed moderate correlations with Borg scale and the VAS Dyspnoea, with a Pearson’s r around -0.5 (details see Table 4). However, we detected significant negative correlations with all LCQ subscales. Hypothesis 2 cannot be confirmed, since the assumed correlations were only moderate. The correlations are significant and in the right direction, but all of them are only moderate and do not reach a Pearson’s r of 0.6.

**Table 4:** Concurrent validity.

	Leicester Cough Questionnaire		
	Physical	Psychological	Social
**SF-36 Role-Physical index**	.424**	.315**	.374**
**SF-36 Physical Functioning index**	.579**	.466**	.507**
**SF-36 Bodily Pain index**	.429**	.264**	.288**
**SF-36 General Health Perceptions index**	.487**	.412**	.423**
**SF-36 Vitality index**	.475**	.338**	.352**
**SF-36 Social Functioning index**	.435**	.375**	.388**
**SF-36 Role-Emotional index**	.240**	.220**	.243**
**SF-36 Mental Health index**	.254**	.172*	.193**
**SF-36 Standardized Physical Health index**	.560**	.403**	.439**
**SF-36 Standardized Mental Health index**	.218**	.198**	.216**
**Borg scale**	-.538**	-.520**	-.554**
**VAS Dyspnoea**	-.572**	-.533**	-.529**

Notes: Pearson’s correlations,

*p<0.05,

**p<0.01,

***p<0.001. Interpretation guide: LCQ scales: high values = low burden, SF-36 scales: high values = high manifestation of related dimensions’ content, Borg scale: high values = high dyspnoea, VAS Dyspnoea: high values = high dyspnoea.

## Discussion

In this study, we translated the LCQ into German language, and examined its psychometric properties in a sarcoidosis cohort. We had sufficient information to analyse 193 out of 200 included patients, which is an acceptable rate for a validation study. In addition, the overall response rates were high. Only item number 9 had a relatively higher non-response frequency, which can be the result of the specific situation as the question asks for exposure to paints or fumes. This situation does not apply for every patient. Furthermore, we observed floor effects in most of the items which indicates that either the chosen reference time (2 weeks) might be too short or the patients overall show no severe health problems. The lowest response category however represents “Never/not at all” which cannot be differentiated any more.

The internal consistency of the German LCQ was good to excellent, with Cronbach’s Alphas between 0.87 to 0.94 for the three subscales. The high internal consistency of the German LCQ, as well as the moderate concurrent validity compared to general health or respiratory-specific HRQL questionnaires, is in line with published data, after translation into several other languages[15,16,19,22,55] as well as in the original LCQ[11]. In this regard it is astonishing that the Physical Component Summary Score (PCS) of the SF-36 showed a quite high correlation with the LCQ, while the Mental Component Summary Score (MCS) does not. This might indicate that the LCQ does measure HRQL aspects which are not covered by the SF-36 emphasizing the importance not only to use the SF-36, but also the LCQ. Here, a moderate correlation does not necessarily mean moderate validity.

The strengths of this study are the use of up-to-date patient reported outcome methodology, including item response modelling and confirmatory factor analyses, and to reach such a sample size in a specific patient group, which was sufficient for the statistical analyses conducted. The main limitations of this study are floor effects mainly because sarcoidosis patients were included regardless of the presence of cough. The moderate concurrent validity and missing Rasch model fit might therefore be attributable to our cohort and might improve if the German LCQ would be applied in a cohort suffering from more severe cough or higher cough frequencies. Further limitations are the following: Patients answered the questionnaire only once, so we cannot evaluate the test-retest-reliability. Additionally, CFA might have suffered from a insufficient sample size [56]. A validation cohort is missing, especially to confirm the good model fit in the CFA. Furthermore, we do not have objective data on physiological parameters of cough, like cough frequency, spectra or duration, which might show improved concurrent validity of the LCQ. And last, we only included patients with sarcoidosis which limits the generalizability of our results for patients with cough as a symptom of other diseases but demonstrates unequivocally its usefulness for measuring sarcoidosis associated cough.

In conclusion, we validated the German LCQ and could show that the psychometric properties of the German LCQ are acceptable for its general application and good for its use in sarcoidosis studies. We provide evidence for reliability, unidimensionality and internal consistency. However, some limitations might raise concerns. Only a moderate concurrent validity, and no Rasch model fit were detected. Furthermore, we found strong floor effects that indicate that the LCQ has limitations regarding its applicability in an asymptomatic sarcoidosis population. However, the moderate concurrent validity is in agreement with the concurrent validity in various other cohorts. Further testing is needed in order to confirm the discriminatory power between the subscales “Physical”, “Psychological” and “Social”. Disregarding the problem of construct validity, we suggest further investigating external validity and possible scale transformations in order to reach Rasch model fit. Further research with larger sample sizes is needed to investigate test-retest reliability and Rasch model fit. The German LCQ is valid, reliable, and ready to use in clinical settings as well as in pharmacological trials.

## Supporting information

S1 Table Items analysis: German version of the Leicester Cough Questionnaire (LCQ). (DOCX)

S2 Table Item difficulty. (DOCX)
