# Supplementary material for: Psychometric properties of the German version of the Leicester Cough Questionnaire in sarcoidosis
Source: PLoS One. 2018 Oct 4;13(10):e0205308. doi: 10.1371/journal.pone.0205308 (PMC6171952; doi:10.1371/journal.pone.0205308)
Supplement: S1 Table — (DOCX) [file pone.0205308.s001.docx]

**S1 Table. Items Analysis: German version of the Leicester Cough Questionnaire (LCQ)**

| **Items** | | **Non-response** | | **Mean values** | | **Item difficulty** | **Skew-ness** | **Kurtosis** | **Distribution of extreme values** | |
| --- | --- | --- | --- | --- | --- | --- | --- | --- | --- | --- |
|  |  | N | % | M | SD | ID | S | K | %  undermost extreme  (7 = never / none at all) | %  upmost extreme  (1 = always / a huge amount) |
| 1 | In the last 2 weeks, have you had chest or stomach pains as a result of your cough?  *Hatten Sie in den vergangenen 14 Tagen Brust- oder Bauchschmerzen aufgrund Ihres Hustens?* | 1 | 0.5 | 5.93 | 1.52 | 0.60 | -1.45 | 1.39 | 54.7 | 2.1 |
|  |  |  |  |  |  |  |  |  |  |  |
| 2 | In the last 2 weeks, have you been bothered by sputum (phlegm) production when you cough?  *Wurden Sie in den vergangenen 14 Tagen durch Auswurf (Schleim) beim Husten gestört?* | 0 | 0.0 | 5.70 | 1.64 | 0.56 | -1.05 | -0.11 | 49.2 | 1.0 |
|  |  |  |  |  |  |  |  |  |  |  |
| 3 | In the last 2 weeks, have you been tired because of your cough?  *Waren Sie während der vergangenen 14 Tage aufgrund Ihres Hustens müde?* | 0 | 0.0 | 5.92 | 1.56 | 0.45 | -1.40 | 0.99 | 56.5 | 1.6 |
|  |  |  |  |  |  |  |  |  |  |  |
| 4 | In the last 2 weeks, have you felt in control of your cough?  *Hatten Sie in den vergangenen 14 Tagen das Gefühl, Ihren Husten unter Kontrolle zu haben?* | 1 | 0.5 | 5.60 | 1.86 | 0.33 | -1.25 | 0.28 | 5.2 | 47.4 |
|  |  |  |  |  |  |  |  |  |  |  |
| 5 | How often during the last 2 weeks have you felt embarrassed by your coughing?  *Wie oft während der vergangenen 14 Tage war Ihnen Ihr Husten unangenehm?* | 1 | 0.5 | 5.49 | 1.88 | 0.67 | -0.99 | -0.32 | 48.4 | 4.2 |
|  |  |  |  |  |  |  |  |  |  |  |
| 6 | In the last 2 weeks, my cough has made me feel anxious  *In den vergangenen 14 Tagen hat mir mein Husten Sorgen gemacht.* | 2 | 1.0 | 5.87 | 1.67 | 0.43 | -1.39 | 0.74 | 57.6 | 1.6 |
|  |  |  |  |  |  |  |  |  |  |  |
| 7 | In the last 2 weeks, my cough has interfered with my job, or other daily tasks  *In den vergangenen 14 Tagen hat mein Husten mich bei meiner Arbeit oder bei anderen täglichen Verrichtungen gestört.* | 1 | 0.5 | 5.91 | 1.63 | 0.89 | -1.38 | 0.71 | 58.9 | 1.6 |
|  |  |  |  |  |  |  |  |  |  |  |
| 8 | In the last 2 weeks, I felt that my cough interfered with the overall enjoyment in my life  *In den vergangenen 14 Tagen hatte ich das Gefühl, dass mein Husten meine allgemeine Lebensfreude beeinträchtigt hat.* | 1 | 0.5 | 6.03 | 1.61 | 0.54 | -1.67 | 1.74 | 63.5 | 2.6 |
|  |  |  |  |  |  |  |  |  |  |  |
| 9 | In the last 2 weeks, exposure to paints or fumes has made me cough  *In den vergangenen 14 Tagen musste ich husten, wenn ich Farben (Lacken) und Dämpfen ausgesetzt war.* | 6 | 3.1 | 5.22 | 2.12 | 0.44 | -0.74 | -0.90 | 50.8 | 9.1 |
|  |  |  |  |  |  |  |  |  |  |  |
| 10 | In the last 2 weeks, has your cough disturbed your sleep?  *Hat Ihr Husten in den vergangenen 14 Tagen Ihren Schlaf gestört?* | 0 | 0.0 | 6.01 | 1.58 | 0.54 | -1.52 | -1.18 | 61.1 | 1.6 |
|  |  |  |  |  |  |  |  |  |  |  |
| 11 | In the last 2 weeks, how many times a day have you had coughing bouts?  *Wie oft am Tag (innerhalb 24 Stunden) hatten Sie in den vergangenen 14 Tagen Hustenanfälle?* | 5 | 2.6 | 5.68 | 1.51 | 0.76 | -0.94 | -0.80 | 43.1 | 1.1 |
|  |  |  |  |  |  |  |  |  |  |  |
| 12 | In the last 2 weeks, my cough has made me feel frustrated  *In den vergangenen 14 Tagen war ich wegen meines Hustens frustriert.* | 0 | 0.0 | 6.21 | 1.37 | 0.49 | -1.78 | 2.25 | 66.8 | 0.5 |
|  |  |  |  |  |  |  |  |  |  |  |
| 13 | In the last 2 weeks, my cough has made me feel fed up  *In den vergangenen 14 Tagen hatte ich den Husten einfach satt.* | 5 | 2.6 | 5.80 | 1.86 | 0.58 | -1.38 | 0.55 | 61.2 | 4.8 |
|  |  |  |  |  |  |  |  |  |  |  |
| 14 | In the last 2 weeks, have you suffered from a hoarse voice as a result to your cough?  *Hat der Husten in den letzten 14 Tagen bei Ihnen Heiserkeit verursacht?* | 1 | 0.5 | 6.14 | 1.43 | 0.66 | -1.60 | 1.51 | 64.6 | 0.5 |
|  |  |  |  |  |  |  |  |  |  |  |
| 15 | In the last 2 weeks, have you had a lot of energy?  *Waren Sie in den vergangenen 14 Tagen voller Energie (Tatendrang)?* | 0 | 0.0 | 4.11 | 1.83 | 0.72 | -0.10 | -1.14 | 8.8 | 8.8 |
|  |  |  |  |  |  |  |  |  |  |  |
| 16 | In the last 2 weeks, have you worried that your cough may indicate serious illness?  *Haben Sie sich in den vergangenen 14 Tagen Sorgen darum gemacht, dass Ihr Husten auf eine ernsthafte Erkrankung zurückzuführen sein könnte?* | 2 | 1.0 | 5.95 | 1.60 | 0.73 | -1.61 | 1.72 | 57.1 | 3.1 |
|  |  |  |  |  |  |  |  |  |  |  |
| 17 | In the last 2 weeks, have you been concerned that other people think something is wrong with you, because of your cough?  *Waren Sie in den vergangenen 14 Tagen besorgt, dass andere Menschen wegen Ihres Hustens denken könnten, mit Ihnen wäre etwas nicht in Ordnung?* | 0 | 0.0 | 6.11 | 1.65 | 0.61 | -1.86 | 2.20 | 67.9 | 3.1 |
|  |  |  |  |  |  |  |  |  |  |  |
| 18 | In the last 2 weeks, my cough has interrupted conversation or telephone calls  *In den vergangenen 14 Tagen wurden meine Unterhaltungen oder Telefongespräche durch Husten unterbrochen* | 0 | 0.0 | 5.91 | 1.64 | 0.74 | -1.46 | 1.18 | 58.5 | 3.1 |
|  |  |  |  |  |  |  |  |  |  |  |
| 19 | In the last 2 weeks, I feel that my cough has annoyed my partner, family or friends  *In den vergangenen 14 Tagen hatte ich das Gefühl, mein Husten würde meine(n) Partner(in), Familie oder Freunde stören.* | 4 | 2.1 | 5.98 | 1.68 | 0.69 | -1.65 | 1.67 | 62.4 | 3.7 |
|  |  |  |  |  |  |  |  |  |  |  |
