# Supplementary material for: Psychometric properties of the German version of the Leicester Cough Questionnaire in sarcoidosis
Source: PLoS One. 2018 Oct 4;13(10):e0205308. doi: 10.1371/journal.pone.0205308 (PMC6171952; doi:10.1371/journal.pone.0205308)
Supplement: S2 Table — (DOCX) [file pone.0205308.s002.docx]

| **Item Difficulty Index values** | | | | | |
| --- | --- | --- | --- | --- | --- |
|  | N | Min | Max | Mean | SD |
| LCQ 01_p | 192 | .02 | 1.00 | .7650 | .30438 |
| LCQ 02_p | 193 | .02 | 1.00 | .7187 | .32838 |
| LCQ 03_p | 193 | .02 | 1.00 | .7653 | .31256 |
| LCQ 04_p | 192 | .02 | 1.00 | .7115 | .34381 |
| LCQ 05_p | 192 | .02 | 1.00 | .6871 | .35608 |
| LCQ 06_p | 191 | .02 | 1.00 | .7610 | .32628 |
| LCQ 07_p | 192 | .02 | 1.00 | .7673 | .32339 |
| LCQ 08_p | 192 | .02 | 1.00 | .7951 | .31335 |
| LCQ 09_p | 187 | .02 | 1.00 | .6474 | .39109 |
| LCQ 10_p | 193 | .02 | 1.00 | .7862 | .31315 |
| LCQ 11_p | 188 | .02 | 1.00 | .7038 | .31050 |
| LCQ 12_p | 193 | .02 | 1.00 | .8259 | .28331 |
| LCQ 13_p | 188 | .02 | 1.00 | .7574 | .34869 |
| LCQ 14_p | 192 | .02 | 1.00 | .8095 | .29419 |
| LCQ 15_p | 193 | .02 | 1.00 | .4132 | .30648 |
| LCQ 16_p | 191 | .02 | 1.00 | .7750 | .31009 |
| LCQ 17_p | 193 | .02 | 1.00 | .8178 | .31238 |
| LCQ 18_p | 193 | .02 | 1.00 | .7666 | .32085 |
| LCQ 19_p | 189 | .02 | 1.00 | .7870 | .32067 |

**S2 Table. Item difficulty**
